# Impaired Intestinal Permeability of Tricellular Tight Junctions in Patients with Irritable Bowel Syndrome with Mixed Bowel Habits (IBS-M)

**DOI:** 10.3390/cells12020236

**Published:** 2023-01-05

**Authors:** Karem Awad, Christian Barmeyer, Christian Bojarski, Oliver Nagel, In-Fah M. Lee, Michal R. Schweiger, Jörg-Dieter Schulzke, Roland Bücker

**Affiliations:** 1 Clinical Physiology, Charité—Universitätsmedizin Berlin, Campus Benjamin Franklin, 12203 Berlin, Germany; 2 Department of Gastroenterology, Infectious Diseases and Rheumatology, Charité—Universitätsmedizin Berlin, Campus Benjamin Franklin, 12203 Berlin, Germany; 3 Institute for Translational Epigenetics, Medical Faculty, University of Cologne, 50931 Cologne, Germany

**Keywords:** irritable bowel syndrome, intestinal barrier function, tight junctions, tricellulin, occludin, leaky gut, RNA-seq

## Abstract

Background: The underlying pathophysiology of irritable bowel syndrome (IBS) is still unclear. Our aim was to investigate the pathophysiological mechanisms of diarrhea, constipation, and antigen uptake in mixed-type IBS (IBS-M). Methods: Colonoscopic biopsies were obtained from IBS-M patients. Epithelial transport and barrier function of colonic mucosae were characterized in Ussing chambers using impedance spectroscopy. Mucosal permeability to macromolecules was measured. Western blotting for tight junction (TJ) proteins was performed and their subcellular localization was visualized by confocal microscopy. RNA-sequencing was performed for gene expression and signaling pathway analysis. Results: In IBS-M, epithelial resistance and ENaC-dependent sodium absorption were unchanged, while short-circuit current reflecting chloride secretion was reduced. Concomitantly, epithelial permeability for fluorescein and FITC-dextran-4000 increased. TJ protein expression of occludin decreased, whereas claudins were unaltered. Confocal microscopy revealed the de-localization of tricellulin from tricellular TJs. Involved pathways were detected as proinflammatory cytokine pathways, LPS, PGE2, NGF, and vitamin D. Conclusions: Decreased anion secretion explains constipation in IBS-M, while ion permeability and sodium absorption were unaltered. Reduced occludin expression resulted in the delocalization of tricellulin from the tricellular TJ, leading to increased macromolecular permeability that contributes to antigen influx into the mucosa and perpetuates a low-grade inflammatory process.

## 1. Introduction

Irritable bowel syndrome (IBS) has a prevalence of 10–20% [1], and is a functional disorder of the digestive system characterized by chronic abdominal pain and altered bowel habits [2]. Four types of IBS are distinguished on the patient’s reported predominant bowel habit: IBS-D with predominant diarrhea, IBS-C with predominant constipation, IBS-M with mixed bowel habits, and unclassified IBS-U [3,4]. Although it is widespread in the world population [5], its pathophysiology has not yet been fully understood. A growing understanding of the underlying mechanisms will contribute to the search for a therapeutic approach. 

One aspect of intestinal function in IBS is an increased permeability to macromolecules, for which a central role in pathophysiology is discussed. Increased permeability to macromolecules means that proteins or lipopolysaccharides (LPS), which represent luminal antigens, can enter the organism more easily and can promote mucosal and/or systemic inflammatory responses. This concept of a barrier disturbance leading to inflammation has been termed the *leaky gut* concept. Responsible for the increased intestinal permeability for macromolecules is an impaired epithelial barrier function, which is a complex result of various components including mucus, the epithelial cell layer with its cell-cell junctions, and the innate immune system [6]. Antigens can cross the intestinal epithelium either transcellularly (via transcytosis) or paracellularly (through disturbed tight junctions (TJ)). A multiprotein complex of claudins and tight junction-associated MARVEL proteins (TAMPs) form the TJ strands [6]. These are highly dynamically regulated structures that close the intercellular space (gate function) and thereby control the flow of molecules through the epithelium. Moreover, the TJs maintain the polarity of the epithelial cells by separating the apical from basolateral membrane compartments, thereby limiting the lateral membrane diffusion of their membrane proteins (fence function). Functionally important protein components of the TJ are occludin, tricellulin, and the claudin family with 27 different claudins present in mammals [7] with either a barrier- or channel-forming function. Most of the claudins are considered to have a tightening function as claudin-1, claudin-3, claudin-4, claudin-5, or claudin-8, while others have a channel-forming function as claudin-2 or claudin-15 [8,9,10,11,12]. Occludin is thought to have a tightening effect, but its function is not essential for ionic permeability, since occludin-deficient mice showed no measurable electrical resistance change of the intestinal epithelium. However, occludin is more important for macromolecule permeability [13]. A specialized function is ensured by tricellulin, which tightens the tricellular tight junctions (tTJs) against the passage of macromolecules. Its disruption has functional relevance in inflammatory bowel disease (IBD), being reduced in ulcerative colitis [14], and being dislocated due to the reduction of angulin-1 in Crohn’s disease [15]. In general, it was proposed that the molecular correlate of impaired barrier function is associated with a change in the composition of epithelial TJ proteins. Moreover, it has been suggested that intestinal barrier dysfunction is associated with visceral hypersensitivity in patients with IBS [16]. Mast cell activation gives an example of how barrier dysfunction results in visceral hypersensitivity as a result of a disrupted epithelial barrier [17]. A previous clinical study has shown that small bowel permeability for ^51^Cr-EDTA (339 Da) is increased in IBS-D and post-infectious IBS when compared to controls [18].

Up to now, however, little is known about intestinal barrier function in the IBS-M subgroup and even less about the colonic transport and barrier function in these patients. Moreover, the expression pattern of TJ proteins in IBS-M has so far not been elucidated. In IBS-M patients, constipation as the predominant phase is interrupted by diarrheal episodes. This IBS-pattern represents the most abundant subtype, reported in over 30% of IBS patients fulfilling Rome III criteria [19].

We expect that IBS-M could have a uniform phenotype. According to our observations, patients with IBS-M have long periods of constipation, which are interrupted from time to time by episodes of diarrhea (“liquid stool overflow”, see discussion).

Therefore, in IBS-M we aimed to characterize (i) paracellular macromolecule permeability in colonic biopsies, (ii) ion permeability as measured with impedance spectroscopy, (iii) the expression pattern of TJ proteins, and (iv) the subcellular localization of barrier relevant TJ proteins using confocal microscopy and super-resolution microscopy. Furthermore, (v) RNA-sequencing (RNA-seq) with bioinformatics pathway analysis was carried out to gain insights into the involved signaling mechanisms in IBS-M.

## 2. Materials and Methods

### 2.1. Subjects

Sigmoid colon biopsies were taken from seven patients with IBS-M (six female, one male) and eleven controls (seven females, four male). IBS-M was diagnosed according to the Rome III criteria, the following diagnostic criteria had to be fulfilled:

Recurrent abdominal pain or discomfort at least 3 days/month in last 3 months associated with two or more of criteria:

- Improvement with defecation
- Onset associated with a change in stool frequency
- Onset associated with a change in stool form (appearance)

In all IBS-M patients, no gross lesions were seen macroscopically or microscopically. None of the patients was on medication at the time of the biopsy. The average age was 30 ± 1 years (controls: 49 ± 3 years).

In control patients, colonoscopy was performed, e.g., for prevention of colon cancer. All biopsies were taken 30 cm ab ano. Specimens were inserted into miniaturized Ussing chambers (exposed tissue area 0.049 cm^2^) as described previously [20]. The time between taking the biopsy and mounting it into the Ussing chamber was about 30 min.

### 2.2. Ethics

For the use of human material, this study adhered to the Declaration of Helsinki. The study was approved by the institutional review board of “The Ethics Committee of the Charité” under the approval number EA4/015/13, and written informed consent was obtained from each patient.

### 2.3. Impedance Spectroscopy

Impedance spectroscopy was performed as described earlier to discriminate epithelial (R^epi^) and subepithelial (R^sub^) resistance contribution to the transepithelial resistance (R^t^) of the colon specimens in Ussing-chamber experiments [21]. 48 discrete frequencies of a viable sine-wave alternating current of 35 mA/cm^2^ were applied ranging from 1.3 to 65 kHz. The changes in tissue voltage were detected by phase-sensitive amplifiers. The calculated values for the complex impedance were plotted in a Nyquist diagram after correction of the resistance of the bathing solution and the frequency behavior of the measuring device for each frequency. Results included the acquiring of R^t^ at low frequencies and R^sub^ at high frequencies. R^epi^ = R^t^ − R^sub^, where R^epi^ reflects the barrier function of net epithelial resistance by TJs. 

The bathing solution for Ussing chamber experiments comprised of (in mmol/L): 140.0 Na^+^, 123.8 Cl^−^, 5.4 K^+^, 1.2 Ca^2+^, 1.2 Mg^2+^, 2.4 HPO_4_^2−^, 0.6 H_2_PO_4_^−^, 21.0 HCO_3_^−^, 10.0 D(+)-glucose, 10.0 D(+)-mannose, 2.5 glutamine, 0.5 β-OH-butyrate, and the antibiotics piperacillin (50 mg/L) and imipenem (4 mg/L) for bacterial growth control during the Ussing experiment. The solution was equilibrated at 37 °C with carbogen gas (95% O_2_ and 5% CO_2_) to a pH of 7.4. The antibiotics did not affect resistance or short-circuit current (Isc).

Tissue viability was checked at the end of each Ussing experiment. For this purpose, active electrogenic chloride secretion was maximally stimulated by prostaglandin E2 (PGE2, 10^−6^ mol/L), theophylline (10^−2^ mol/L), and carbachol (10^−4^ mol/L). At the end, bumetanide (10^−5^ mol/L) was serosally added to quantify rheogenic chloride secretion.

### 2.4. Fluorescein and FITC-Dextran-4000 Flux Studies

The permeability for the paracellular flux marker fluorescein was determined from tracer flux measurements, which were performed in Ussing chambers under voltage-clamped conditions. Fluorescein (fluorescein sodium salt; Sigma-Aldrich, Darmstadt, Germany) was added apically at 100 µM concentration. Samples were collected basolaterally after 15, 30, 45, and 60 min. In parallel experiments, 0.4 mM of the dialyzed FITC-labeled dextran-4000 (FITC-dextran with 4 kDa) was added to the apical side and unlabeled 4 kDa dextran was added to the basal compartment, and then samples were collected every 30 min from the basal side. Analysis of fluorescence was made in duplicate in a plate reader at 535 nm (Tecan SpectroPhotometer, Tecan Infinite M200, Tecan, Männedorf, Switzerland) using calibration of fluorescence from defined dilutions. Permeability for fluorescein or FITC-dextran was calculated from P = J/Δc with P = permeability (cm/s), J = flux (mol⋅h^−1^⋅cm^−2^), and c = concentration (mol/L).

### 2.5. Western Blot Analysis

Western blot analysis was performed on colonic biopsies. A lysis buffer with 20 mM Tris (pH 7.4), 5 mM MgCl_2_, 1 mM EDTA, 0.3 mM EGTA, and complete protease inhibitor mixture (Roche AG, Basel, Switzerland) facilitated protein extraction and homogenization by passing through a needle (27.5 G). The extract centrifugation for the removal of insoluble material occurred at 200× *g* for 5 min at 4 °C, the supernatant was then centrifuged at 43,000× *g* for 30 min at 4 °C. The pellet (membrane fraction) was resuspended in lysate buffer. Separation of 20 µg protein was performed with polyacrylamide gel electrophoresis and transfer to a PDVF membrane (Perkin Elmer, Rodgau, Germany). The development of blots involved the use of primary rabbit (rb) polyclonal IgG antibodies against claudin-1, -2, -3, -5, -8, tricellulin, and occludin (rb claudin-1 #519000, rb claudin-2 #516100, rb claudin-3 #341700, rb claudin-5 #341600, rb claudin-8 #710222, rb tricellulin #700191, rb occludin #711500; all: Invitrogen, Karlsruhe, Germany) and the primary mouse monoclonal IgG antibodies against claudin-4 and β-actin (Sigma-Aldrich). Protein expression was quantified by densitometry with luminescent imaging (LAS-1000, Fuji Film, Tokyo, Japan) using AIDA software (Raytest, Berlin, Germany).

### 2.6. Immunohistochemistry and Confocal Laser-Scanning Microscopy (LSM)

Immunofluorescence microscopy is used to precisely visualize structures in tissues and cells using antibodies. In this work, the subcellular localization of TJ proteins in the sigmoid colon was analyzed by immunofluorescence. First, sigmoid biopsies were fixed in 1% paraformaldehyde for 60 min at room temperature. They were then rinsed in PBS+Ca/Mg and incubated in 25 mM glycine for five minutes. After they were washed a second time in PBS+Ca/Mg, the biopsies were successively dehydrated by an increasing sucrose series (10%, 20%, and 30%). For this purpose, the tissue was incubated for 60 min each at room temperature. To prepare the cryoblocks, the biopsies were frozen in methylbutane chilled with liquid nitrogen, then placed in TissueTek (Sakura Finetek, Europe B.V, Umkirch, Germany) and subsequently stored at −80 °C. Tissue sections of 5 μm thickness were prepared by a cryostat (Leica CM 1950, Leica, Wetzlar, Germany). These were frozen on coated slides at −20 °C for 12–24 h prior to staining. The tissue sections were permeabilized with the 0.5% Triton-X solution for 10 min at room temperature. Subsequently, unspecific binding was blocked with blocking solution for 60 min (1% Goat serum, 5% BSA, 0.05% Triton X- 100). Finally, overnight incubation took place with the primary antibody (occludin #711500/#331500, or tricellulin #700191; each 1:100, Invitrogen, Karlsruhe, Germany) at 4 °C. After the first incubation step, three washing steps followed and then incubation was performed with the fluorescent secondary antibody Alexa Fluor-488 goat anti-rabbit (#A32731) and Alexa Fluor-594 goat anti-mouse (#A32742, 1:500, Invitrogen) for 120 min at 37 °C. After each incubation, a rinse with blocking solution was performed. To visualize the nuclei, tissue sections were stained with 4′-d-diamidino-2-phenylidole dihydrochloride (DAPI, 1:1000, Roche AG) and then washed with PBS+Ca/Mg. After nuclei staining, the tissue sections were washed with Aqua bidest. and ethanol. Then, they were finally mounted with Pro Tags Mount Fluor (Biocyc GmbH & Co KG, Potsdam, Germany) on glass slides.

Confocal laser-scanning microscopy (Zeiss LSM 780, Zeiss, Jena, Germany) was used on fluorescence-labeled tissue sections to localize the subcellular distribution of the TJ proteins. According to the secondary antibodies, the excitation wavelengths 594 nm (red) and 488 nm (green) as well as 358 nm (blue for DAPI) were used. Images were obtained with 40× and 63× NA 1.4 plan apochromat plan neofluor objectives. LSM imaging software was used to evaluate and record the stainings (Zeiss, Jena, Germany). To visualize and determine the main localization of tricellulin, we performed an intensity plot analysis with the ZEN blue 2.5 lite software and the embedded software tool “profiles” (Zeiss LSM ZEN software, Carl Zeiss Microscopy GmbH, Jena, Germany). The intensity of tricellulin was measured in the tTJ and was put into relation to the intensity of tricellulin in the bicellular tight junction (bTJ) at a distance of 2 µm away from the measure point of three cells in the tTJ. The measurements were performed on 3 controls in comparison to 3 patients with IBS-M. Measurements were taken 3 times in each of 3–7 sections for each patient.

For stimulated emission depletion (STED) microscopy, the primary antibodies against occludin and tricellulin above mentioned were used in colonic tissue slides as described accordingly and then stained with secondary antibodies Aberrior STAR RED and Aberrior STAR ORANGE (1:200; Abberior GmbH, Göttingen, Germany) for 100 min at 37 °C. After incubation, the tissue slides were washed (3× PBS, 1× Aqua dest.) and mounted with Mounting medium (25 µL, Abberior GmbH, Göttingen, Germany). A STED–compatible cover slide (Carl Zeiss, Jena, Germany) was placed on each slide. TJ protein localization was visualized using a STED microscope (STED Abberior Facility Line, Abberior GmbH, Göttingen, Germany).

### 2.7. Morphometry of the Mucosal Surface Area

Since transport and barrier parameters depend on mucosal architecture, mucosal surface area was calculated in IBS-M and control. For these measurements, the tissues were fixed with 4% formalin directly after Ussing experiments and were then embedded in paraffin at the same degree of stretch as in the electrophysiological experiments. H- and E-sections (hematoxylin & eosin) were prepared and examined by light microscopy in low-power fields (10× magnification). Morphometry was performed on digitally stored light micrographs with Image J software. Morphometry was used to determine the inner crypt diameter in cross sections of the tissues, crypt length, and the number of crypts per serosal area.

### 2.8. Apoptosis Staining

Morphological evaluations were performed on biopsies fixed in 4% formalin. Paraffin-embedded specimens were serially sectioned. Terminal deoxynucleotidyl transferase-mediated deoxyuridine triphosphate nick-end labeling (TUNEL, In Situ Cell Death Detection Kit, Fluorescein, Roche AG) stained cellular DNA fragments. Apoptotic nuclei and all DAPI-positive nuclei were counted to determine the apoptosis rate.

### 2.9. Next-Generation Sequencing

For differential gene expression analysis, we used the technique of RNA-seq followed by bioinformatics pathway analysis. Total RNA was obtained from the sigmoid biopsies using the mirVanaTM RNA isolation kit (Ambion, Life Technologies, Carlsbad, CA, USA). RNA sequencing was performed using the TrueSeq Stranded Total RNA method on an Illumina NovaSeq 6000 sequencing system with RNA quality values of ≥80%. RNA sequences obtained from RNA-Seq were mapped against the human genome GRCh38 release 97 and sorted using STAR aligner version 2.7.1a in a two-pass mode. In first-pass read mapping, coordinates from Ensembl Annotation Release 97 were used as frames. In the second-pass mapping, splice sites found in the first pass were added. Gene-count tables with gene-read coverages were created using the Counts Function of the Bioconductor package Rsubread with coordinates from the Ensembl annotation mentioned above and default parameters. The Bioconductor package DESeq2 was used to quantify the differential expressions of genes between two states in terms of log2-fold changes with their corresponding *p* values. Pathway analysis and upstream regulator analysis were performed using Ingenuity Pathway Analysis software (IPA, Qiagen Silicon Valley, Redwood, CA, USA). Fastq files containing the raw unprocessed sequencing data and a raw data matrix table are deposited on the European Genome-Phenome Archive (EGA) database under record number EGAD00001006646, accessed on 8 March 2021 (https://ega-archive.org/datasets/EGAD00001006646) accessed on 8 March 2021.

### 2.10. Statistical Analysis

For statistical analysis, GraphPad Prism software, version 7.0 (GraphPad Software Inc., San Diego, CA, USA) was used. All values represent mean and SD (standard deviation). Student’s *t*-test was used to calculate significances. The significant level of *p* < 0.05 was used and denoted as: n.s. = not significant, * = *p* < 0.05, ** = *p* < 0.01, *** = *p* < 0.001.

## 3. Results

### 3.1. Active Chloride Secretion Is Diminished in the Colon of IBS-M Patients

**Figure 1:**
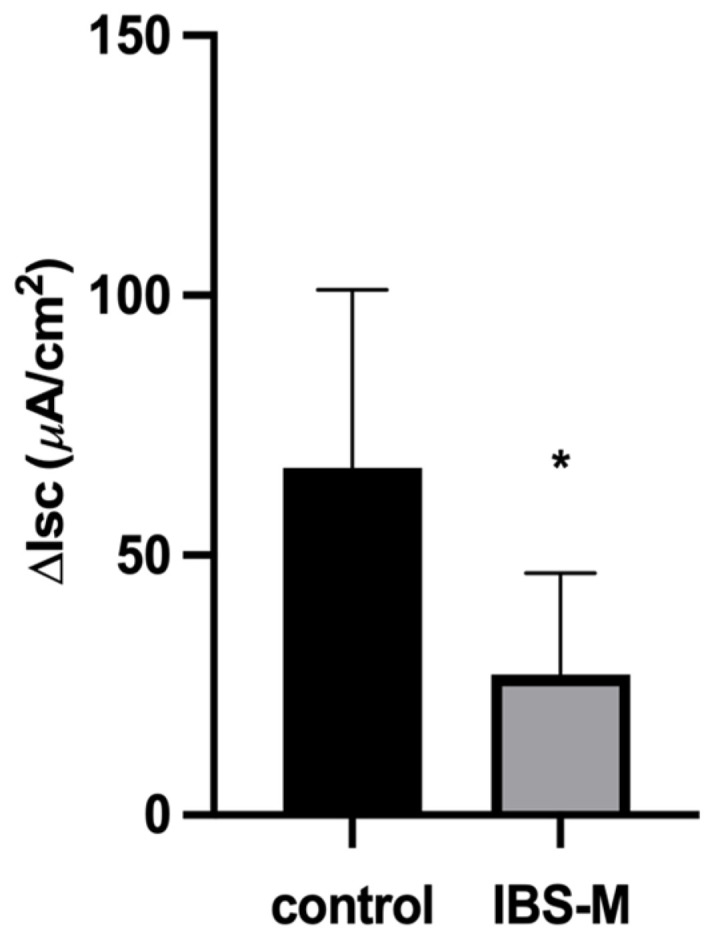
Active electrogenic chloride secretion is diminished in IBS-M. Short-circuit current (Isc, in µA·cm^−2^) of sigmoid colon mucosa from controls and patients with irritable bowel syndrome (IBS-M). Baseline Isc represents the basal Isc at the beginning of the experiment and was 138 ± 75 (n = 10) in control and 74 ± 35 (n = 6) in IBS-M (*p* = 0.0839). Bumetanide (10 µM) was added to the serosal side of the chamber to block the Na-K-Cl cotransporter (NKCC1). ΔIsc represents the difference between the maximum Isc before and the Isc 20 min after addition of bumetanide. Data represent means ± SD with the number of patients as n, n.s. = not significantly different from control, * *p* < 0.05. *p*-Value = 0.0216.

Mucosal biopsy specimens from the distal colon were studied in Ussing chambers for transport and barrier function. In controls, short-circuit current (Isc) amounted to 138 µA·cm^−2^ indicating a significant ongoing active electrogenic transport activity (Figure 1). In the large intestine under baseline conditions, active anion secretion is the source of this Isc, i.e., active electrogenic secretion of chloride and/or bicarbonate [22]. In contrast to the control, Isc in IBS-M was only 74 µA·cm^−2^ with a reduction in active chloride secretion, which becomes evident when chloride secretion was quantified by inhibition with bumetanide (Figure 1), reflecting the constipation condition of IBS-M [23]. In parallel to these functional measurements, we found molecular evidence for a dysfunction of active chloride transport in RNA-sequencing (RNA-seq) data, which revealed a reduced mRNA expression of transporters involved in active chloride secretion as NKCC1, CLCA1, and CFTR (Table 1).

### 3.2. Unaltered Epithelial Sodium Channel Transport Function in IBS-M

**Figure 2:**
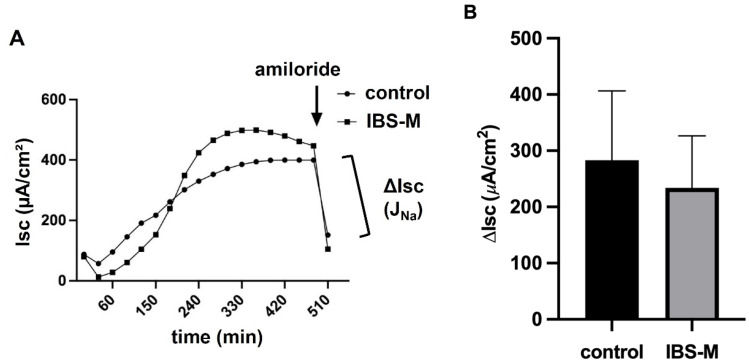
Electrophysiological measurements in the colon mucosa from IBS-M patients. (**A**) Electrogenic Na^+^ absorption in IBS-M. Sodium transport J_Na_ after stimulation by aldosterone was determined as the decrease in short-circuit current Isc after adding amiloride, a selective ENaC channel blocker. Representative data from two separate experiments, squares represent values from a patient with IBS-M and circles from a healthy control. (**B**) Colon biopsies of 8 controls and 7 patients with IBS-M were measured for ENaC-dependent electrogenic Na^+^ transport in miniaturized Ussing chambers, with no statistical significance between the groups in Student’s *t*-test. Data represent mean ± SD.

Since sodium malabsorption in the colon is a possible diarrheal mechanism or, conversely, increased sodium absorption would contribute to constipation, we examined the activity of the epithelial sodium channel (ENaC), which is the rate-limiting transport protein of sodium uptake in the distal colon and rectum. Biopsy samples from IBS-M patients and controls were examined in Ussing chambers during 6–8 h stimulation with 3 nM aldosterone. Electrogenic sodium absorption (J_Na_) was quantified as a decrease in Isc after the addition of 10^−4^ mol/L amiloride (Figure 2A). Results were presented as mean ± SD of eight controls and seven IBS-M patients, respectively. However, no difference was observed between controls and patients with IBS-M (Figure 2B). Thus, the ENaC-dependent uptake of sodium in the distal colon of IBS-M patients was not affected. Consistent with the functional finding shown in Figure 2B, there were no changes in gene expression (Table 2) in our RNA-seq data from colonic mucosae of IBS-M patients and controls in the ENaC subunits α, β, and γ (SCNN1A, SCNN1B, SCNN1G, all not significant; sequencing data are deposited under EGAD00001006646).

### 3.3. Unaltered Ion Permeability in IBS-M Patients in Impedance Spectroscopy

**Figure 3:**
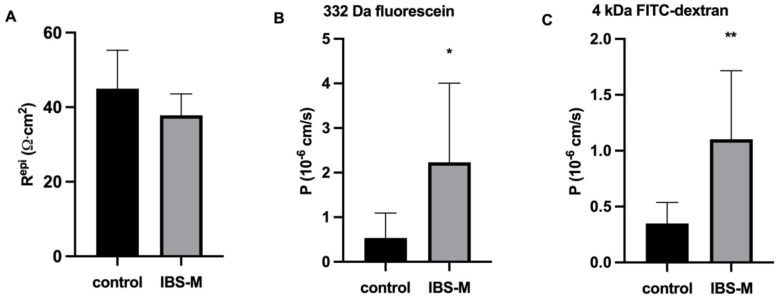
Epithelial resistance and colonic permeability for macromolecules in IBS-M. (**A**) Epithelial barrier function. In miniaturized impedance Ussing chamber for measuring small endoscopic colon biopsies, transmural electrical resistance, subepithelial resistance, and epithelial resistance (R^epi^) were discriminated by alternate current impedance spectroscopy. R^epi^ was not reduced in patients with IBS-M. Controls: n = 11; IBS-M: n = 7; n.s. (**B**) For IBS-M (n = 7; * *p* < 0.05), 332 Da fluorescein showed increased permeability compared with control (n = 10). (**C**) FITC-dextran-4000 (4 kDa), IBS-M (n = 8; ** *p* < 0.01) showed increased permeability compared with control (n = 8). Statistical comparison was performed using Student’s *t*-test for unpaired samples. Data represent mean ± SD.

Ion permeability or conductance (the reciprocal of the electrical resistance) of the colon mucosa was characterized, since it can influence stool consistency with an increase of ion permeability leading to diarrhea (along a leak flux mechanism) and vice versa. Impedance spectroscopy was used to characterize pure epithelial resistance in biopsy specimens in the Ussing chamber, even if additional submucosal series resistances were present and altered in IBS. However, epithelial resistance (R^epi^) turned out to be unchanged in IBS-M when compared to the control (Figure 3A). That R^epi^ was not reduced in IBS-M fits well with the constipation of these patients.

### 3.4. Increased Macromolecule Permeability in IBS-M Patients

In contrast to ion permeability, tracer flux measurements revealed a 4-fold increase in permeability for 332 Da fluorescein (Figure 3B) and a 3-fold increased macromolecular permeability for 4 kDa FITC-dextran in IBS-M colon biopsies (Figure 3C). This is direct evidence for the opening of a leak pathway in IBS-M patients allowing the entry of antigens into the colon mucosa.

### 3.5. Tight Junction Protein Expression: Occludin Is Reduced in IBS-M

**Figure 4:**
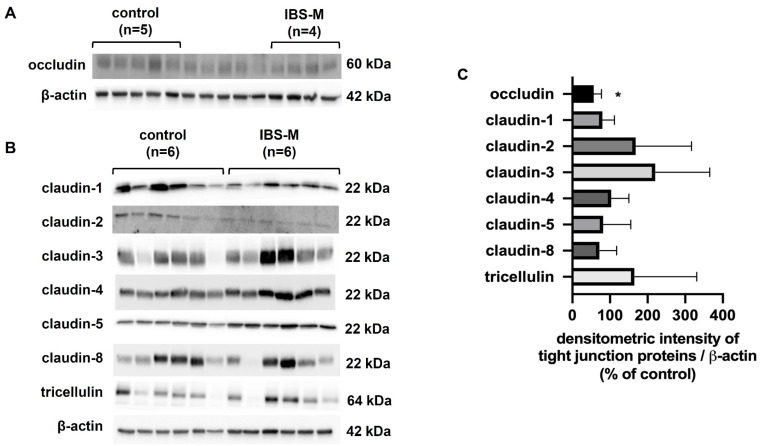
Western blotting and densitometry of TJ proteins. (**A**) Protein expression in colon mucosae by Western blot for occludin (five controls and four IBS-M patients). The five unlabeled lanes in the middle of the Western blot belong to a different inflammatory control group and can be ignored for the present IBS-M study. (**B**) Western blots for tricellulin, claudin -1, -2, -3, -4, -5, and -8 (n = 6 each group). (**C**) Densitometric analysis of Western blots from control subjects (n = 5–6) and patients with IBS-M (n = 4–6). The expression of proteins from control subjects was set to 100%. * *p* < 0.05 when comparing control and IBS-M in Student’s *t*-test. Data represent mean ± SD.

In order to identify the structural correlate of the increase in macromolecule permeability in IBS-M, we studied the expression of strand-forming TJ proteins in Western blots. Occludin, claudin-1, claudin-2, claudin-3, claudin-4 claudin-5, claudin-8, tricellulin, and ZO-1 were examined. However, the quantity of most of the TJ proteins was unaltered in IBS-M. In Figure 4A,B, Western blots are presented, the densitometry of which is depicted in Figure 4C. As the only change occludin expression was found to be decreased in IBS-M, while all other TJ proteins showed an unaltered expression (Figure 4C).

### 3.6. Redistribution of Tricellulin off the Tricellular Tight Junction in IBS-M

**Figure 5:**
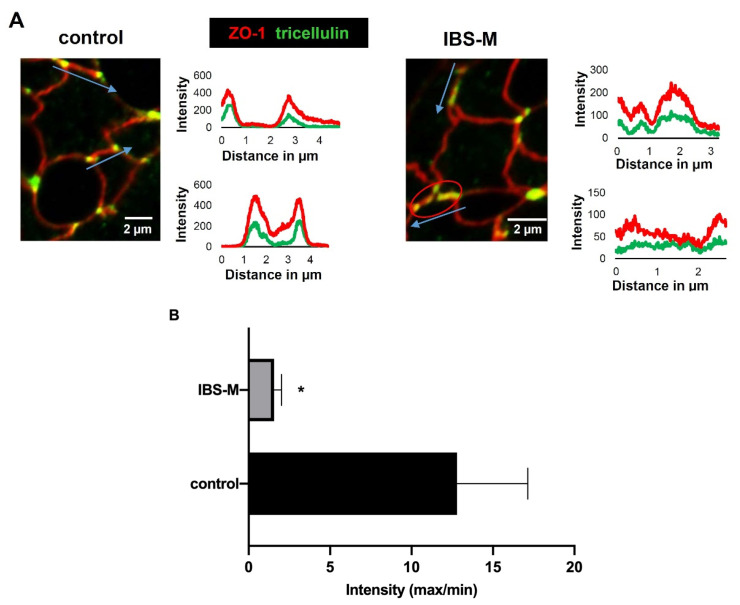

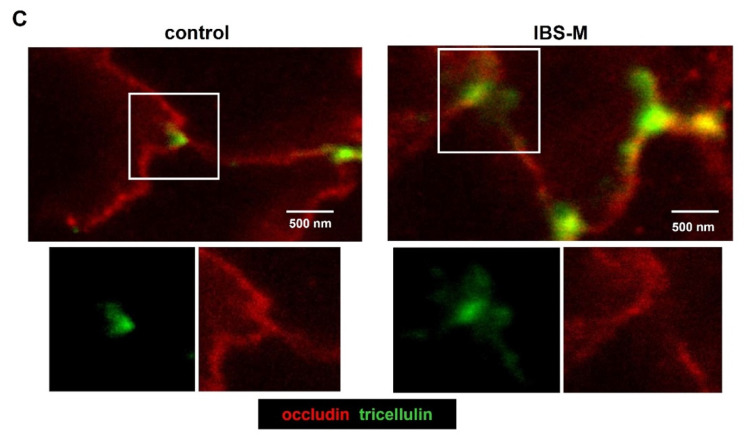
Immunofluorescence detection of tight junction proteins in IBS-M patients with confocal microscopy and super-resolution microscopy. (**A**) Shown are colonic crypts from a control biopsy and IBS-M biopsy. Tricellulin is stained green, ZO-1 is stained red, and the co-localization of tricellulin and ZO-1 appears yellow (merge). Overall, tricellulin staining in the TJ appears to be attenuated in IBS-M compared to control (red signals predominate in IBS-M). The red circle (right) indicates a sorting out from the tricellular tight junction (tTJ). Distance-intensity plots visualize the de-localization of tricellulin away from tTJs. In control, tricellulin (green) shows defined signal peaks at the tricellular meeting points of the TJ as indicated by the blue arrows (marked are length and direction of the pixel intensity measurement along the TJ). In contrast, the intensity plot in IBS-M shows no peak but rather disseminated signals, indicating redistribution of tricellulin from the tricellular to the bicellular TJ. (**B**) The intensity of tricellulin was measured in the tTJ in relation to the intensity in the bicellular tight junction (bTJ) at a distance of 2 µm from the measuring point in the tTJ. A low-intensity ratio (tTJ/bTJ) indicates that tricellulin is sorted out of the tTJ which was more pronounced in IBS-M patients than in controls (each n = 3 patients; * *p* < 0.05). (**C**) Super-resolution stimulated emission depletion (STED) microscopy of occludin (red) and tricellulin (green) in patients’ colon mucosa crypts. In controls, tricellulin is restricted to tTJs (bar = 500 nm). In contrast in IBS-M, the tricellulin signal was redistributed into the bTJ. Data represent mean ± SD.

In addition to the quantitative determination of TJ proteins in the Western blot, we determined the subcellular localization of strand-forming TJ proteins to identify whether a change in distribution with a consequent loss of a TJ protein from the TJ strands could contribute to the epithelial barrier impairment. Therefore, immune-stained tissue sections were examined by confocal laser-scanning microscopy. Interestingly, a significant alteration in tricellulin localization was detected as the most important finding, namely a redistribution off the tTJ (Figure 5A), where it is important for tightening against a passage of macromolecules. This is supported by intensity plots comparing tricellulin immunofluorescence in tricellular and bicellular epithelial TJs (Figure 5A,B). Furthermore, confocal microscopy analysis of tricellulin and ZO-1 showed less co-localization in IBS-M compared to the control. In addition, a decrease in microscopic occludin fluorescence intensity signal was observed in IBS-M compared to the control, corresponding to the reduced expression of occludin in Western blots. With super-resolution STED microscopy, it was possible to visualize the delocalization of tricellulin away from the tTJ in IBS-M (Figure 5C).

### 3.7. RNA-Seq Analysis of IBS-M

To follow up on changes that might play a role in epithelial transport and barrier function of the colon in IBS-M, RNA-seq data from patients’ colonic biopsies were analyzed. First, mRNA expression of various TJ proteins was considered but not found to be altered: for claudin-1, claudin-3, claudin-4, claudin-5, claudin-7, and claudin-8 (Table 3). No change in RNA expression was detected for tricellulin and occludin either (Table 3). That the decrease in protein expression of occludin was not paralleled by a decrease in mRNA level indicates post-transcriptional regulation and/or accelerated protein degradation.

### 3.8. Ingenuity Pathway Analysis of IBS-M

RNA-seq data were further analyzed by bioinformatics pathway analysis tool (Ingenuity Pathway Analysis IPA software), which predicts activated or inhibited signaling pathways based on the mRNA expression pattern of pathway-related genes (upstream regulator analysis). This indicated cytokines and lipopolysaccharides (LPS) as outlined in Table 4 to be effectors in IBS-M with the most activated signaling along their downstream target genes. This points to a mucosal immune response and has to be assumed to contribute to the functional defects in IBS-M.

Beyond cytokine signaling in Table 4, further pathways were found to be affected in the IPA data point to changes in epithelial transport function and visceral hypersensitivity in the mucosa as e.g., prostaglandin E2 (PGE2) (z-score = 0.59, *p*-Value of overlap = 2.70∙e^−3^) and nerve growth factor (NGF) (z-score = 1.89, *p*-Value of overlap = 3.51∙e^−4^) (see Supplemental Table S1).

Other signaling pathways related to micronutrients and functional food components such as curcumin and quercetin were also affected (curcumin: z-score = 0.06, *p*-Value of overlap 4.75∙e^−2^; quercetin: z-score = 0.76, *p*-Value of overlap = 1.49∙e^−3^). This means that such diet components could achieve beneficial regulatory effects. In addition, calcitriol (active vitamin D)-dependent regulation was activated (z-score = 1.03, *p*-Value of overlap = 1.81∙e^−4^) (Supplemental Table S1). This might be of importance for IBS patients with an imbalance in vitamin D status. However, these kinds of bioinformatics predictions provide only a hint and the predictions need to be confirmed experimentally as performed in the present paper for barrier function.

### 3.9. Epithelial Architecture and Apoptotic Rate Remained Unchanged in IBS-M

**Figure 6:**
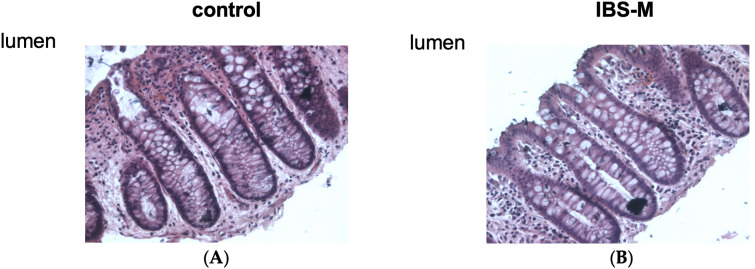
Histological analysis of the sigmoid colon of patients with IBS-M. Representative sections of biopsy specimens from the sigmoid colon of (**A**) controls and (**B**) IBS-M, stained with H&E and visualized with a 20× microscope objective. No differences were seen between controls and IBS-M regarding epithelial architecture.

Epithelial resistance/conductance and transepithelial tracer fluxes are usually referred to impaired epithelial TJs but can also be altered by changes in mucosal architecture (e.g., more TJ area is exposed in the chamber when the mucosal surface area is increased). Furthermore, an increase in the rate of epithelial apoptosis or epithelial gross lesions can affect barrier function. However, no erosions or ulcers were detected in conventional histology in our IBS-M mucosae (data not shown). Morphological analysis did not show any difference in the surface area of the mucosa in IBS-M patients either (ratio of mucosal surface area to serosal area was 4.57 ± 0.58 in control, and 4.97 ± 0.70 in IBS-M (n = 5 each; n.s.) (Figure 6). Finally, in TUNEL-stained sections epithelial apoptotic ratio was unaltered in IBS-M with an epithelial apoptotic rate of 1.1% ± 0.6% compared to 1.0% ± 0.7% in control (n = 5 each; n.s.) 

## 4. Discussion

In the present study, we aimed for a deeper characterization of the barrier properties and TJs in IBS-M. This is important because such pathological changes can explain altered bowel habits, such as diarrhea (along a leak flux diarrhea mechanism) or constipation. On the other hand, they could also explain the route of antigen entry into the mucosa that perpetuates IBS by triggering immune reactions in the mucosa. Since IBS patients are a fairly heterogeneous group with potentially different barrier pathologies, we defined our study group as precisely as possible and included only mixed-type IBS patients (IBS-M) outside of a diarrheal episode. IBS-M patients often present with constipation on the first presentation, but others also present with meteorism and/or abdominal pain. The constipation phase, which dominates in IBS-M, can last up to several weeks. According to our observations, the constipation is interrupted by phases of diarrhea in the sense of a “liquid stool overflow”, which occurs when hard stool clumps together and cannot be easily removed.

We found a decrease in active anion secretion indicated by less bumetanide-sensitive rheogenic transport in the IBS-M colon samples, consistent with the constipation phase of these IBS-M patients. In our accompanying mRNA-seq analysis of these IBS-M biopsy samples, this decrease in active anion secretion in the Ussing chamber was accompanied by a lower expression of components of the active chloride secretion system, namely NKCC1 and CFTR, supporting and in part explaining the finding of a functional downregulation of active anion secretion in the colon of our patients. In contrast, active sodium absorption via the ENaC was not affected. Furthermore, ionic barrier function, measured as electrical resistance of the epithelium, R^epi^, was found to be intact, which is important for the ability of an epithelium to absorb sodium against steep gradients and to concentrate the feces in the distal colon. At this point, it should be mentioned that the constipation in our IBS-M patients is occasionally interrupted by episodes of diarrhea, which could be interpreted as a purging event to prevent intestinal obstruction. During these diarrheal phases, active chloride secretion may be transiently activated, but this was not examined in the present study. In addition, other mechanisms that contribute to IBS symptomatology must also be assumed. For example, the PGE2 pathway was found to be activated in our IPA pathway analysis. On the one hand, this could be a mechanism that leads to episodes of diarrhea in IBS-M patients via anion secretion [24,25]. On the other hand, PGE2 plays a central role in the visceral hypersensitivity of IBS patients [26]. Visceral hypersensitivity is also an important criterion for the diagnosis of IBS and in the focus of research on the pathophysiology of IBS. With NGF another mediator of visceral hypersensitivity was identified in our IPA analysis, which additionally revealed vitamin D deficiency as a factor in susceptibility to IBS-M and proposed curcumin and quercetin as potential functional foods for IBS-M patients.

As an important finding of our study, barrier function was disturbed for macromolecules in IBS-M. Having the unchanged electrical resistance of the epithelium in mind, this may be surprising at first view. A possible explanation is that macromolecule channels, which are much less frequent than permeability sites for ions, become more abundant in IBS-M. This significantly affects macromolecule fluxes but causes only a tendency for the electrical resistance to decrease. A similar explanation had been proposed for differentially altered lactulose over monosaccharide permeability ratios in celiac disease [27,28]. How important the careful definition of the IBS subgroup criteria in such types of analyses is, becomes apparent from a study, in which 4 kDa FITC-dextran permeability of the duodenum and colon was found to be unaltered in female IBS-C patients [23]. That a barrier defect for macromolecules with low-grade immune activation exists in IBS-patients with diarrheal episodes but is absent in permanent constipation (IBS-C) is exciting, since it could be an important surrogate marker and a different pathomechanism and thus needs further attention and experimental confirmation. An increase in macromolecule permeability is assumed to promote mucosal inflammation. Disruption of the epithelial barrier function allows antigens to enter the mucosa, leading to an immunological response in the subepithelium. Thus, endotoxins such as lipopolysaccharides or lipooligosaccharides (LPS or LOS) as macromolecules could play a role in the immunologic response in IBS-M. This immune response, in turn, can compromise the integrity of the mucosa, leading to epithelial leakage, which in turn facilitates antigen penetration. This creates a vicious cycle.

In recent years, our understanding of the importance of intestinal barrier function and epithelial TJ alterations for the pathogenesis of barrier disorders considerably increased, e.g., with a reduced expression of occludin and claudin-4 in collagenous colitis [29], and of claudin-5 and claudin-8 in Crohn’s disease, as well as an increase in the channel-forming TJ protein claudin-2 in ulcerative colitis [30]. As an important regulatory mechanism in these inflammatory conditions as well as in many infectious intestinal diseases, a strong mucosal cytokine release has been identified to influence barrier function and TJ integrity, which in turn allows antigens to enter the mucosa as a trigger for the immune system (leaky gut phenomenon) [20,31,32,33].

In the present study, the TJ protein expression of several claudins present in the large intestine was checked and found to be unchanged in IBS-M, which fits with the unaltered epithelial resistance. In contrast, the expression of occludin was decreased, a TJ protein discussed to be involved in the tightening of the epithelial TJ against macromolecules [34]. Furthermore, the subcellular localization of tricellulin was detected to be changed in confocal microscopy, and in super-resolution microscopy, namely, tricellulin was segregated from the tTJ into the bTJ. This is another explanation for the increase in macromolecule permeability since reduced tricellulin expression and/or subcellular redistribution of tricellulin off the tTJ has been directly shown to increase macromolecule permeability [14,35]. In support of our interpretation, Ikenouchi and co-workers reported that occludin knock-down causes mislocalization of tricellulin to bTJs, implying that occludin supports tricellular localization of tricellulin by excluding tricellulin from bTJs [36]. As a consequence of the higher uptake of 332 Da fluorescein (9 Å) and 4 kDa dextran (14 Å), a subclinical low-grade inflammation has to be assumed. Indeed, we directly found activation of cytokine pathways of TNFα, IL-1β, IFNγ, and other cytokines by RNA-seq and IPA pathway analysis in our IBS-M patients. Previous studies also detected higher cytokine release as e.g., IL-1β in colorectal specimens of IBS patients [37,38]. Thus, it may be reasonable to conclude that the subclinical cytokine release within the mucosa leads to altered TJ protein expression and/or localization with barrier impairment. In previous studies of our group, low-dose cytokine cocktails (TNFα, IL-1β, IFNγ, and IL-13) affected barrier function in HT-29/B6 cells and colon biopsies [33,39]. Furthermore, our group showed a reduction of occludin in response to TNFα in the HT-29/B6 colon cell model [40]. Thus, most of the interactions between immune cells and epithelial cells seem to be mediated by cytokines and more and more pathways have been discovered that target TJ proteins.

In the IPA upstream regulator analysis based on the RNA-seq data, we identified mediators of visceral hypersensitivity including TNFα, which is consistent with previous descriptions [41]. We also identified activated pathways of cytokines (interleukins and interferons) and the main inductor of these pro-inflammatory pathways was LPS. The activation pattern of pro-inflammatory cytokines and LPS pathways resembles the picture of IPA data from colonic specimens of acutely infected *C. jejuni* patients [33], although to a much lower extent (with slighter changes of z-scores and overlap *p*-Values), which reflects the character of a subclinical low-grade immune response in IBS. This low-grade cytokine release might be sufficient to induce the observed barrier effects during chronification of the symptoms. Especially, they might be sufficient to induce the changes in tricellulin localization via occludin expression reduction in IBS-M. Thus taken together, with the tricellulin delocalization, we have presented a mechanistic model for the subclinical inflammatory response of IBS-M patients. The challenge of future approaches to the role of leaky gut phenomena in IBS will be the chicken-and-egg issue. On the one hand, a weakened barrier might enable antigen influx and cytokine release and on the other hand, cytokine release might weaken barrier function. Therefore, further systemic inputs have to be assumed as initial input into the system like dietary metabolites or the microbiota and/or previous infectious events with prolonged low-grade inflammatory responses on the background of a genetic predisposition [42].

## Figures and Tables

**Table 1:** Chloride secretion-related genes in RNA-seq datasets of IBS-M colon.

Gene Name	Expression (Fold Change)	*p*-Value
SLC12A2 (basolateral Na-K-Cl cotransporter, NKCC1)	−1.8 log2	0.0005
CLCA1 (calcium-activated chloride channel regulator 1)	−1.9 log2	0.002
CFTR (cystic fibrosis transmembrane conductance regulator)	−1.4 log2	0.003

Sequencing data are deposited at the European Genome-Phenome Archive (EGA) database under record number EGAD00001006646.

**Table 2:** Gene expression of ENaC subunits in RNA-seq datasets of IBS-M colon.

Gene Name	Expression (Fold Change)	*p*-Value
SCNN1A (α-ENaC)	0.3 log2	0.906
SCNN1B (β-ENaC)	0.4 log2	0.898
SCNN1G (γ-ENaC)	0.2 log2	0.954

The sequencing data are deposited at the European Genome-Phenome Archive (EGA).

**Table 3:** Expression of tight junction genes in RNA-seq datasets of IBS-M colon.

Gene Name	Expression (Fold Change)	*p*-Value
CLDN1 (claudin-1)	0.8 log2	0.826
CLDN3 (claudin-3)	0.6 log2	0.583
CLDN4 (claudin-4)	2.1 log2	0.053
CLDN5 (claudin-5)	−0.7 log2	0.804
CLDN7 (claudin-7)	1.2 log 2	0.286
CLDN8 (claudin-8)	0.7 log2	0.908
OCLN (occludin)	1.2 log2	0.401
MARVELD2 (tricellulin)	0.4 log2	0.832

Sequencing data are deposited at the European Genome-Phenome Archive (EGA).

**Table 4:** Upstream regulator analysis. Top upstream regulators in immune function.

Upstream Regulator	*p*-Value of Overlap	Activation z-Score	Target Molecules
CSF2	4.81∙e^−8^	2.668	CCL23, CDKN1A, DDIT3, GK, IFNLR1, PIM1, PLAUR, PPP1R15A, RHOV, SLC2A1
IFN-γ	8.38∙e^−8^	3.060	ADM, CCL20, CCL23, CCNO, CD274, CDKN1A, CRABP1, DDIT3, DKK1, DUSP5
TNF-α	8.53∙e^−8^	3.979	ADM, CCL20, CD274, CDKN1A, DDIT3, DKK1, DUSP5, EPHA2, IGFBP2, IRF7
LPS	1.45∙e^−7^	3.914	ADM, CCL20, CD274, CDKN1A, CYP3A5, DDIT3, DUSP5, GK, GPC4, IRF7
IL-4	2.29∙e^−7^	2.099	CCL20, CCL23, CD274, CDKN1A, CRABP1, EPHA2, HRH1, IFNLR1, IRF7, MUC5B
IL-1β	5.62∙e^−7^	2.877	ADM, CCL20, CD274, CDKN1A, CYP3A5, DDIT3, DUSP5, ERRFI1, IRF7, MUC5B

Upstream effectors lipopolysaccharides (LPS) and top proinflammatory cytokines (CSF, colony-stimulating factor; IFN, interferon TNF, tumor necrosis factor; IL, interleukin) with significant activation of their downstream targets in colon specimens from patients with IBS-M (n = 3 patients and n = 4 healthy controls). Column 1; Gene name of the upstream regulator. Column 2; *p*-Value of the overlap of the downstream target gene set and the pathway gene set. Column 3; Activation z-score. Column 4; Names of genes with expression changes. Sequencing data are deposited on the European Genome-Phenome Archive (EGA) database under record number EGAD00001006646. RNA-seq data were analyzed with Ingenuity Pathway Analysis (IPA) software. The complete list of genes differentially expressed in patients with IBS-M and controls, which are downstream targets of the upstream regulator is provided in the Supplement (Supplementary Table S1). The overlap *p*-Value measures whether there is a statistically significant overlap between the data set genes and the genes that are regulated by an upstream transcriptional regulator. It is calculated using Fisher’s exact test, and significance is generally attributed to *p*-Values < 0.01. A statistical approach defines a quantity (z-score) that determines whether an upstream transcription regulator has significantly more “activated” predictions than “inhibited” predictions (z > 0) or vice versa (z < 0).

## Data Availability

Fastq files containing the raw unprocessed sequencing data and a raw data matrix table are deposited on the European Genome-Phenome Archive (EGA) database under record number EGAD00001006646, accessed on 8 March 2021 (https://ega-archive.org/datasets/EGAD00001006646) accessed on 8 March 2021.

## Supplementary Materials

The following supporting information can be downloaded at: https://www.mdpi.com/article/10.3390/cells12020236/s1, Table S1: Upstream regulators in IBS-M. Entire list of activated and inactivated pathways in Upstream regulator analysis (IPA software). To expand the list with the affected pathways, chose the filter button in Predicted Activation State tab and Select All.

## Abbreviations

BSA	Bovine serum albumin
bTJ	Bicellular tight junction
CFTR	Cystic fibrosis transmembrane conductance regulator
CLCA1	Chloride channel accessory 1
DAPI	4′-d-diamidino-2-phenylidole dihydrochloride
EDTA	Ethylenediaminetetraacetic acid
EGTA	Ethylene glycol-bis (β-aminoethyl ether)-N,N,N′,N′-tetraacetic acid
ENaC	Epithelial sodium channel
FITC	Fluorescein isothiocyanate
IBD	Inflammatory bowel disease
IBS	Irritable bowel syndrome
IBS-M	mixed-type irritable bowel syndrome
IFN-γ	Interferon-gamma
IL-1β	Interleukin-1 beta
Isc	Short-circuit current
NKCC1	Na-K-Cl cotransporter 1
NGF	Nerve growth factor
LPS	Lipopolysaccharides
PBS	Phosphate-buffered saline
PDVF	Polyvinylidene fluoride
PGE2	Prostaglandin E2
rb	rabbit
R^epi^	Epithelial resistance
R^sub^	Subepithelial resistance
R^t^	Transmural resistance
SD	Standard deviation
STED	Stimulated Emission Depletion
TAMPs	Tight junction-associated MARVEL proteins
TJ	Tight Junction
TNF-α	Tumor necrosis factor-alpha
tTJ	Tricellular tight junction
TUNEL	Terminal deoxynucleotidyl transferase biotin-dUTP nick end labeling
ZO-1	Zonula occludens protein-1
